# Inhibitory Effects of Carbazomycin B Produced by *Streptomyces roseoverticillatus* 63 Against *Xanthomonas oryzae* pv. *oryzae*

**DOI:** 10.3389/fmicb.2021.616937

**Published:** 2021-03-24

**Authors:** Tingting Shi, Xin Guo, Jiali Zhu, Lingming Hu, Zhipeng He, Donghua Jiang

**Affiliations:** College of Chemistry and Life Sciences, Zhejiang Normal University, Jinhua, China

**Keywords:** *Streptomyces roseoverticillatus*, carbazomycin B, *Xanthomonas oryzae* pv. *oryzae*, biological control, antibacterial mechanism

## Abstract

The present manuscript highlights the potential role of *Streptomyces roseoverticillatus* 63 (Sr-63) against *Xanthomonas oryzae* pv. *oryzae* (Xoo), which is the cause of a disastrous bacterial leaf blight disease with rice worldwide. The disease suppression was achieved under greenhouse conditions. A foliar spray of the fermentation broth of Sr-63 significantly reduced the leaf blight symptoms with rice in Xoo inoculated rice plants. Furthermore, we observed that the carbazomycin B, isolated from the fermentation broth of Sr-63, was demonstrated to have antibacterial activity against Xoo with a minimum inhibitory concentration (MIC) of 8 μg mL^–1^. The results indicated that carbzomycin B hampered the membrane formation of Xoo, reduced the production of xanthomonadin and extracellular polymeric substance (EPS). The fourier transform infrared spectroscopic (FT-IR) indicated that carbazomycin B changed the components of the cell membrane, then caused a change of the cell surface hydrophobicity of Xoo. Scanning electron microscopy revealed that the Xoo cells treated with carbazomycin B exhibited apparent structural deformation. The results also indicated that carbazomycin B had a negative impact on the metabolism of Xoo, carbazomycin B reduced the activity of malate dehydrogenase (MDH) activity and suppressed the protein expression of Xoo. Overall, our data suggests that *Streptomyces roseoverticillatus* 63 is a promising biocontrol agent that could be used to combat the bacterial leaf blight diseases of rice.

## Introduction

Plant disease causes dramatic yield losses worldwide (Latz et al., 2016), bacterial leaf blight (BLB) disease caused by *Xanthomonas oryzae* pv. *oryzae* (Xoo) is considered to be one of the most serious bacterial diseases in rice, leading to severe yield losses (Koduru et al., 2020). Generally, this disease can be managed by chemical control, but effectiveness is often strain-dependent due to the overreliance on chemical control methods (Sahu et al., 2018). Microbial biological control agents (MBCAs) are applied to crops for biological control of plant pathogens, where they act via a range of modes of action (Köhl et al., 2019). Production of antimicrobial secondary metabolites, with inhibiting effects against pathogens, is one of the modes of action (Raaijmakers and Mazzola, 2012).

*Actinobacteria* represent the most prominent group of microorganisms that producing bioactive compounds. They synthesize approximately two-thirds of all naturally derived antibiotics currently used in medicine, veterinary practice and agriculture, mostly from *Streptomyces* genus (Barka et al., 2015). A previous study have reported that *Streptomyces bottropensi*, isolated from the soil of Imsil-gun (South Korea), could produce Bottromycin A2 and Dunaimycin D3S antibiotics for antagonized BLB (Park et al., 2011). *Streptomyces* sp. MJM4426 can protect rice leaf explants from the infection of Xoo by producing staurosporine (Cheng et al., 2016). *Streptomyces toxytricini* VN08-A-12 was found, not only able to reduce the length of Xoo lesion in the two rice cultivars (the reduction of lesion length was up to 38.3%), but also significantly reduced the loss of Xoo-related yield in infected rice cultivars from the field (yield loss reduction of up to 43.2%) (Van Hop et al., 2014). Anthracimycin isolated from the *Streptomyces* sp. strain CNH365 is especially effective against *Bacillus anthracis*, and its mode of action is based on the inhibition of DNA/RNA synthesis (Jang et al., 2013). Buanmycin, isolated from the culture of a marine *Streptomyces* strain from a tidal mudflat in Buan (South Korea), inhibits the *S. aureus* sortase A, an enzyme that plays a key role in adhesion and host invasion by Gram-positive bacteria (Moon et al., 2015). Kocurin is a new thiazoleptide obtained from *Kocuria Palustris* F-276345, whose mode of action most likely is to inhibit bacterial growth, by blocking the biosynthesis of its protein at the translation stage (Jakubiec-Krzesniak et al., 2018). Salinamide F showed significant inhibition of Gram-positive and Gram-negative bacterial RNA polymerase (Hassan et al., 2015). Some of natural products of cultured actinomycetes also showed antifungal or antiviral activities (Igarashi et al., 2017; Chen et al., 2018). All antibacterial, antifungal and antiviral agents demonstrate the huge potential of actinomycetes as leader producers of novel bioactive molecules. The majority of the described compounds were discovered by a traditional approach, screening for metabolites in the actinobacterial fermentation broth.

In a previous study, we screened the role of *Streptomyces* strains inhibiting Xoo and demonstrated that the *Streptomyces roseoverticillatus* 63 (Sr-63) had a strong inhibitory action against Xoo (inhibition zone with 46.48 ± 2.32 mm of diameter). We therefore evaluated the biological control of Sr-63 against BLB, identified the antibacterial substance(s) produced by Sr-63, and gained insight into the underlying mechanisms responsible for the antagonistic effect against Xoo.

## Materials and Methods

### Bacterial Strains and Culture Conditions

The Sr-63 was isolated from the rhizosphere soil of *Ophiopogon bodinieri*, at Zhejiang Normal University, Zhejiang Province, China, and sustained on Gauze’s Synthetic Medium No.1 that was prepared with 20 g of soluble starch, 1 g of KNO_3_, 0.5 g of K_2_HPO_4_, 0.5 g of MgSO_4_∙7H_2_O, 0.5 g of NaCl, 0.01 g of FeSO_4_ ∙ 7H_2_O per 1,000 mL of distilled water (pH 7.0–7.2). The Sr-63 strain was deposited at China Center for Type Culture Collection (CCTCC) on April 15th, 2019, with registration number CCTCC M 2019261.

The *Xanthomons oryzae* pv. *oryzae* P6 (Xoo), was provided by the genetics laboratory of the College of Chemistry and Life Sciences of Zhejiang Normal University. The Xoo bacterial suspensions were prepared, cultured with Xoo in a nutrient agar (NA) medium in a shaking incubator at 28°C until OD_600_ = 0.6. The NA medium was prepared with 1 g of yeast extract, 3 g of beef extract, 5 g of polypetone, 10 g of sucrose, 15 g of agar powder per 1,000 mL of distilled water (pH 7.0–7.2). The NB medium contained the same components but excluding agar powder. Unless otherwise stated, the Xoo strain was used for all the experiments.

### Preventative and Curative Effect Experiments

The fermentation broth of Sr-63 was tested *in vivo* against BLB disease. The seeds of rice (Zhonghua 11), obtained from China National Rice Research Institution, were transplanted into the soil after culturing on wet filter water for 3 days at 28°C, and the seedlings were propagated in the greenhouse (28 ± 1°C, 70% relative humidity, and 10/14 light regimen). The fermentation broth of *Streptomyces* strains was prepared by incubating Sr-63 on Gauze’s Synthetic Medium No.1 at 30°C for 5 days with 180 rpm, centrifuging at 12,000 × *g* (Eppendorf, Germany) for 10 min at 4°C, and filtering the supernatant using a 0.22-μm filter. The rice seedlings were inoculated by Xoo bacterial suspensions with sterile cotton, after cutting the leaves when the leaves reached 20 cm (four- or five-leaf stage). The plants were sprayed with the fermentation broth of Sr-63 strain (3 times within 3 h), immediately followed by (Treatment 1, T1), 6 h (Treatment 2, T2), 12 h (Treatment 3, T3), and 24 h (Treatment 4, T4) until the broth dripped off the plants, respectively. Spraying plants on Gauze’s Synthetic Medium No.1 only were used as controls (CK). The treatment was repeated for three plants with three leaves each. The distance from the tip to the leading edge of grayish to chlorotic tissue was taken as a measure of the progression of blight disease. Disease symptoms were measured and recorded 14 days later. The best effect of control treatment was compared with the commercial agent bismerthiazol (500 μg mL^–1^).

Lesion⁢inhibition⁢ratio=(CK-T)/CK×100%

### Fermentation, Extraction, Purification, and Identification of Antibacterial Compound Produced by Sr-63

The Sr-63 strain was cultured on Gauze’s Synthetic Medium No.1 (fifty 1,000 mL Erlenmeyer flasks, each containing 400 mL of Gauze’s Synthetic Medium No.1) for 7 days in a shaker rotating (28°C, 160 rpm). The total cultured broth was extracted with EtOAc at room temperature. The EtOAc phase was evaporated *in vacuo* R-300 (Buchi, Switzerland) to provide a crude extract (21.65 g) and then chromatographed on silica gel (SiO_2_; 100–200 mesh; Qingdao Marine Chemical Ltd., Qingdao, China) with a step gradient of petroleum ether (PE)/ethyl acetate (EA) (100% PE→ 100% EA, 1 mL min^–1^) to give the fractions. Fractions with biological activity against Xoo were further loaded onto a Sephadex LH-20 (column 140 × 2 cm, MeOH, 0.5 mL min^–1^) to give the pure active compound (Zhang et al., 2008). Fractions were monitored by thin-layer chromatography (TLC) and observed under ultraviolet (UV) light at 254 and 365 nm.

^1^H and ^13^C nuclear magnetic resonance (NMR) spectra were obtained on a 600 MHz spectrometer (Bruker AVANCE, Switzerland) using chloroform-d (CDCl)_3_ as the solvent at room temperature, while tetramethylsilane was used as an internal standard. Chemical shifts (δ) are given in parts per million, and coupling constants (J) are in hertz (Hz). Signal patterns are indicated as follows: s, singlet; d, doublet; dd, doublet of doublets; dt, doublet of triplets; ddd, doublet of doublets of doublets; t, triplet; m, multiplet; bs, broad singlet, quin., quintet. Mass spectrum analyses were performed by using an Agilent 6230 TOF LC-MS spectrometer (Agilent Technologies, Santa Clara, CA, United States). A drying gas flow rate of 8 L min^–1^ using a temperature of 300°C and a capillary voltage of 3.5 kV.

### Minimum Inhibitory Concentration

For stock solutions, carbazomycin B was dissolved in filter-sterilized (0.22 μm) dimethyl sulfoxide (DMSO) at a concentration of 2,560 μg mL^–1^. The stock solutions were diluted with distilled water immediately before use. Minimum inhibitory concentration (MIC) values for the test were determined by microdilution in 96-well plates as recommended by the Clinical and Laboratory Standards Institute (CLSI, 2020) with some modification. The medium used was NB medium. Briefly, approximately 5 × 10^5^ CFU mL^–1^ of Xoo was inoculated into NB medium containing 2-fold dilutions of the carbazomycin B (concentration range from 0.25 to 128 μg⋅mL^–1^). The same procedure was performed with the following controls: NB without carbazomycin B; NB containing 10^5^ CFU mL^–1^ of Xoo (positive control); NB containing 10^5^ mL^–1^ of Xoo and bismerthiazol (0.25–128 μg⋅mL^–1^, negative control). The plates were incubated for 24 h at 37°C to allow for bacterial growth, and the optical density was calculated at 600 nm (OD_600_) using a microplate reader (Thermo Fisher Scientific, Madison, WI, United States), to assess the growth of bacterial culture. The exact same experiment was repeated three times.

### Pathogenicity Assays

The Xoo cells grown up to the log phase were centrifuged and resuspended to 1 × 10^8^ CFU mL^–1^ with 0.1 M phosphate-buffered solution (PBS, pH = 7.4), then carbazomycin B (4 × MIC) was added at 28°C for 4 h. Xoo cells treated with PBS for 4 h were used as the positive control. The Xoo bacterial suspensions were inoculated with rice plants Zhonghua 11 as described above. Plants inoculated with PBS were used as the negative control. The distance from the tip to the leading edge of grayish to chlorotic tissue was taken as a measure of the progression of blight disease. Disease symptoms were monitored and recorded 14 days later. Then a repeat treatment of three plants with three leaves each.

### Effect on Biofilm Formation

Biofilm formation was quantified as described previously (Abdallah et al., 2019). Carbazomycin B solutions were prepared by adding carbazomycin B stock solutions to an NB medium. 10 μl of Xoo suspension (1 × 10^8^ CFU mL^–1^) was added to 90 μl of NB in 96-well plates, and 100 μl of carbazomycin B was added to each well to give a final carbazomycin B concentration of 1/2 × MIC, 1 × MIC, 2 × MIC, and 4 × MIC. Wells without carbazomycin B were used as the controls. Plates were incubated at 28°C, the culture medium was poured out after 24 h, and attached bacterial cells were gently washed three times with distilled water. The cells were then stained with 1% crystal violet (200 μl) for 30 min, and incubated for 25–30 min at room temperature. Unbound crystal violet was poured out, and the wells were washed three times with water. Biofilm formation was achieved by adding 150 μl of 33% acetic acid, and its absorbance was measured at 570 nm, using a spectrophotometer (Thermo Fisher Scientific, Madison, WI, United States). Three replicates were used for quantitative measurement.

### Scanning Electron Microscopy

Scanning electron microscopy was used to determine the effect of carbazomycin B on morphological changes in Xoo. The Xoo cells grown up to the log phase were further diluted to adjust its final concentration to 1 × 10^8^ CFU mL^–1^, and then treated with various concentrations of carbazomycin B (1/2 × MIC, 1 × MIC, 2 × MIC, and 4 × MIC) for 4 h at 28°C. The bacterial suspensions were centrifuged at 12,000 × *g* (Eppendorf, Germany) for 10 min. Xoo cells were fixed with 2.5% glutaraldehyde overnight at 4°C, after washing three times with 0.1 M phosphate-buffered solution (PBS, pH = 7.4). Fixed cells were rinsed three times for 10 min with the same buffer, and dehydrated through an ethanol gradient [30, 50, 70, 90, 100% (v/v)]. For SEM analysis, samples were dried in a benchtop freeze drier (BMH, Beijing, China) for 24 h, coated with gold in a Hitachi ion sputterer E1030 (Hitachi, Japan), and then analyzed on a Gemini scanning electron microscope 300 (Carl Zeiss Microscopy GmbH, 73447 Oberkochen, Germany). This experiment was performed in triplicates.

### Fourier Transform Infrared Spectroscopic (FT-IR) Analysis

The Xoo cells were further diluted to adjust its final concentration to 1 × 10^8^ CFU mL^–1^, and treated with various concentrations of carbazomycin B (MIC and 2 × MIC) for 4 h at 28°C. The untreated cells were used as the control. The Xoo cells were harvested by centrifugation (Eppendorf, Germany) at 1,000 × *g* for 10 min, pellets were placed in a benchtop freeze drier (BMH, Beijing, China) for 24 h, after washing three times with 0.1 M phosphate-buffered solution (PBS, pH = 7.4). Fourier transform infrared spectroscopic (FT-IR) spectra of Xoo cells with and without carbazomycin B were obtained in transmission mode in wavenumber range 500–4,000 cm^–1^, using Nicolet iS5 Thermo Scientific spectrophotometer (Thermo Fisher Scientific, United States), and the variations in the components of cell membrane were determined using OMNIC software 7.3. For each spectrum, 16 scans were collected at a resolution of 4 cm^–1^. Each sample was scanned with three different pellets, under identical conditions. The average of three replicates was used to analyze the spectra, using ORIGIN 6.0 software.

### Effect on the Hydrophobicity of Xoo Cells

The method of microbial adhesion to hydrophobicity (MATH) (Rosenberg, 2006) was used to measure the effect of carbazomycin B on cell surface hydrophobicity of Xoo. The Xoo cells were diluted to adjust its final concentration to 1 × 10^8^ CFU mL^–1^, and treated with various concentrations of carbazomycin B (1/2 × MIC, 1 × MIC, 2 × MIC, and 4 × MIC) for 4 h at 28°C. The control was prepared without the addition of carbazomycin B. The Xoo cells were centrifuged, and then suspended with 0.1 M phosphate-buffered solution (PBS, pH = 7.4) to a corrected absorbance at 400 nm of 0.4 (A_0_). The cell suspension (4 mL) was placed in tubes and 1 mL of n-hexadecane added. The mixtures were agitated uniformly in a vortex mixer for 120 s, and kept at room temperature to allow for the separation of the two phases for 15 min; the OD of the aqueous phase was determined at 400 nm (A1) using an ultraviolet spectrophotometer UV-7504 (Xinmao, Shanghai, China). The percentage of hydrophobicity was calculated as follows: (A_0_−A_1_)/A_0_ × 100% (Yamanaka-Okada et al., 2008). This experiment was performed in triplicates.

### Determining the Influence of Carbazomycin B on the Metabolism of Xoo

#### Effect on the Production of Extracellular Polymeric Substance

To measure the effect of carbazomycin B on the production of extracellular polymeric substance (EPS), the Xoo suspensions (1 × 10^8^ CFU mL^–1^, 1 mL) were inoculated in NB medium (99 mL) containing final concentrations of carbazomycin B (1/2 × MIC, 1 × MIC, 2 × MIC, and 4 × MIC), and maintained at 28°C, 160 rpm for 24 h. Control was prepared without the addition of carbazomycin B. After incubation, 10 mL portions of the culture were collected by centrifugation (Eppendorf, Germany) at 8,000 × *g* for 20 min at 4°C (Guo et al., 2010). Three volumes of ethyl alcohol were added to the supernatants, and the mixture was refrigerated overnight (at 4°C) for the precipitation of EPS. The precipitated EPS were pelleted via centrifugation, dried, and weighed (Vojnov et al., 1998). This experiment was performed in triplicates.

#### Quantification of Xanthomonadin

Measurement of xanthomonadin pigment was based on the method as described by Sahu et al. (2018), with some modification. Briefly, the Xoo cells, treated with carbazomycin B (1/2 × MIC, 1 × MIC, 2 × MIC, and 4 × MIC) for 4 h were collected by centrifuging 4 mL broth suspension and mixing with 2 mL 100% methanol. The untreated cells were used as the control. The mixtures were further incubated in darkness for 10 min, kept on a rotating shaker, and followed by centrifugation (Eppendorf, Germany) at 10,000 × *g* for 10 min to collect the supernatant. The xanthomonadin pigment was estimated by measuring the absorbance at OD_445_ using an ultraviolet spectrophotometer UV-7504 (Xinmao, Shanghai, China). This experiment was repeated three times.

#### Metabolic Enzyme Malate Dehydrogenase Activity

The Xoo cells grown up to the log phase were centrifuged and resuspended to 1 × 10^8^CFU mL^–1^ with phosphate-buffered solution (PBS), then the Xoo was added with carbazomycin B using final concentrations of 1 × MIC, 2 × MIC, and 4 × MIC for 4 h at 28°C, with PBS as a control. Then, 10 mL of each culture was centrifuged (Eppendorf, Germany) at 10,000 × *g* for 10 min, and resuspended in 1 mL of 0.1 M phosphate-buffered solution (PBS, pH = 7.4). The bacteria were disrupted by a digital sonifier (Branson ultrasonic corporation, Danbury, United States). The protein content, malate dehydrogenase (MDH) activity was determined using a commercial assay kit (Nanjing Jiancheng Bioengineering Institute, China), according to the manufacturer’s recommendations. The MDH activity was expressed in U/mg protein. The test was performed three times, independently.

#### Sodium Dodecyl Sulfate Polyacrylamide Gel Electrophoresis Analysis

A total of 10 mL of Xoo cells suspensions (1 × 10^8^ CFU mL^–1^) were added with carbazomycin B at final concentrations of 1 × MIC, 2 × MIC, and 4 × MIC for 4 h at 28°C. The Xoo cells were harvested by centrifugation (Eppendorf, Germany) at 1,000 × *g* for 10 min and resuspended with distilled water and SDS-PAGE sample loading buffer. The bacterial suspension was centrifuged, after boiling for 10 min, then an SDS-PAGE analysis was performed using 10% polyacrylamide gels. Gels were stained with Coomassie brilliant blue R-250. The test was performed three times, independently.

### Statistical Analysis

SPSS.20 and ANOVA were used to analyze the obtained data, and the Duncan test was used to evaluate significant differences between the treatments. Significant differences were identified by analyzing the comparison between the control and the treated samples using the *post hoc* test (^∗^*p* < 0.05, ^∗∗^*p* < 0.01, and ^∗∗∗^*p* < 0.001).

## Results

### Preventative and Curative Effect

To evaluate the practical feasibility of using Sr-63 against Xoo, the fermentation broth of Sr-63 was tested *in vivo* against BLB disease. The results were shown in Table 1 (Supplementary Figure 1). The result showed that the fermentation broth of Sr-63 had remarkable curative activity against BLB, with a control effect of 95.35% under the T1, while the control effect of bismerthiazol was 91.87%. The results also indicated the good curative control of fermentation broth of Sr-63 on Xoo infected rice plants, and the earlier treatment with Sr-63 fermentation broth gave better suppression effects.

**TABLE 1:** The lesion length suppression effect of fermentation broth from *Streptomyces roseoverticillatus* 63 against bacterial leaf blight disease under different treatments.

**Treatment**	**Lesion length (cm)**	**Suppression ratio (%)**
Control (CK)	117e	–
Bismerthiazol	9.5a	91.88a
Treatment 1 (T1)	5.4a	95.38a
Treatment 2 (T2)	27.3b	76.67b
Treatment 3 (T3)	34.5c	70.51c
Treatment 4 (T4)	43.7d	62.65d

*Means in a value followed by the same letter are not significantly different at *P* < 0.05 based on Duncan test.*

### Isolation of Antibacterial Substance From Sr-63

In order to identify the substance activity against Xoo produced by Sr-63, fermentation broth of Sr-63 was used for isolation by EtOA. Finally, the antibacterial substance, carbazomycin B was obtained as a brown crystal. ^1^H NMR and ^13^C NMR data were as follows (Supplementary Figures 2–5): ^1^H NMR (600 MHz, CDCl_3_): δ: 8.28 (d, J = 7.7 Hz, 1H), 7.74 (brs, 1H), 7.40 (m, 2H), 7.25 (m, H), 6.14 (brs, 1H), 3.85 (s, 3H), 2.42 (s, 3H), 2.39 (s, 3H). ^13^C NMR (101 MHz, CDCl_3_): δ: 142.0, 139.3, 138.5, 136.8, 127.0, 124.8, 123.3, 122.7, 119.5, 110.1, 109.4, 109.4, 61.5, 13.2, 12.8. HRESIMES m/z Calcd for C_15_H_15_NO_2_ [M + H]^+^ 242.1176. Found 242.1176; [M + Na]^+^: 264.0995, Found 264.0096. These showed almost no difference compared with carbazomycin B (C_15_H_15_NO_2_), described in the literature (Sakano and Nakamura, 1980).

### Minimum Inhibitory Concentration

To assess the inhibitory activity of the carbazomycin B on Xoo, a broth microdilution assay was used. The results indicated that the MIC value of carbazomycin B against Xoo was 8 μg mL^–1^, while for bismerthiazol the MIC value was found to be 16 μg mL^–1^ (Supplementary Figure 6). The data clearly demonstrated that carbazomycin B has a strong antibacterial activity against Xoo *in vitro* condition.

### Assay of the Effects of Carbazomycin B on Xoo Cell Virulence

**FIGURE 1:**
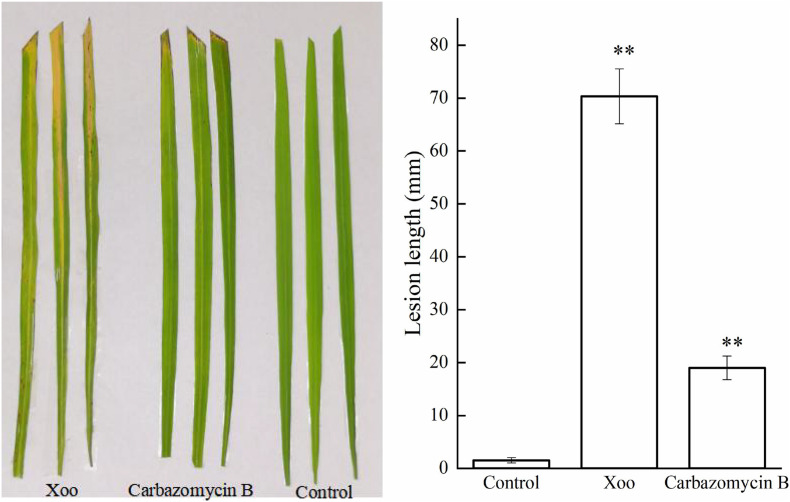
Carbazomycin B reduced the virulence of *Xanthomonas oryzae* pv. *oryzae* (Xoo). The untreated Xoo and Xoo treated with 4 × MIC for 4 h of carbazomycin B were inoculated on rice plants. The lesion length was measured after 14 days of inoculation. Results are means of three technical replicates and the bar indicates the standard deviation. Significant differences were determined by a *post hoc* test (^∗∗^*p* < 0.01).

After ascertaining the inhibitory activity of carbazomycin B against Xoo, we performed an *in vivo* bioassay on the actual rice plants. As shown in Figure 1 the lesion lengths observed in rice plants (Zhonghua 11), inoculated with Xoo cells were significantly reduced (72.98%) after treated with carbazomycin B (4 × MIC). The data indicated that the carbazomycin B reduced the virulence of Xoo. Taken together, this data demonstrated that carbazomycin B has a strong antibacterial activity in both *in vitro* and *in vivo* conditions against Xoo, it can be effectively used as an alternative strategy to control the most devastating bacterial blight disease in the major crop plant rice.

### Carbazomycin B Perturbs the Biofilm Formation by Xoo

**FIGURE 2:**
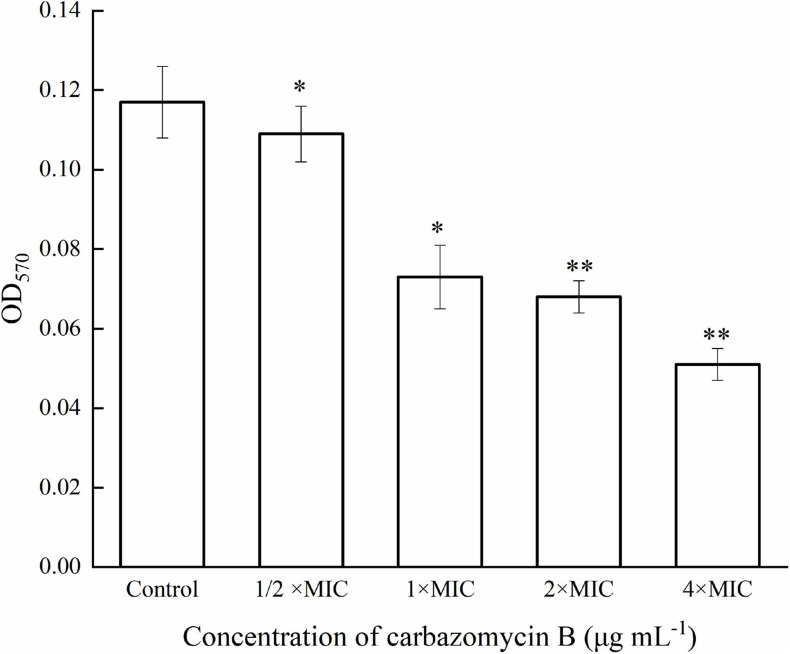
Inhibitory effect of carbazomycin B on biofilm formation of *Xanthomonas oryzae* pv. *oryzae* (Xoo). Results are means of three technical replicates and the bar indicates the standard deviation. Significant differences were determined by a *post hoc* test (^∗^*p* < 0.05 and ^∗∗^*p* < 0.01).

Biofilm refers to the complex communities of microbes, and the biofilm lifestyle allows the bacteria to withstand such hostile environmental conditions, like starvation and desiccation (Roy et al., 2018). In order to gain further insight into the potential inhibitory mechanism of carbazomycin B, crystal violet assay was utilized to monitor the formation of biofilm by Xoo. As expected, carbazomycin B was found to restrict the developmental of biofilm by Xoo. The results indicated that the biofilm formation of Xoo was reduced by cabarzomycin B in the OD_570_ value, compared to the control (Figure 2). The OD_570_ value of Xoo was reduced by 37.6, 41.9, and 56.4% when treated with carbazomycin B at 1 × MIC, 2 × MIC, and 4 × MIC.

### Carbzazomycin B Causes Morphological Changes in Xoo

**FIGURE 3:**
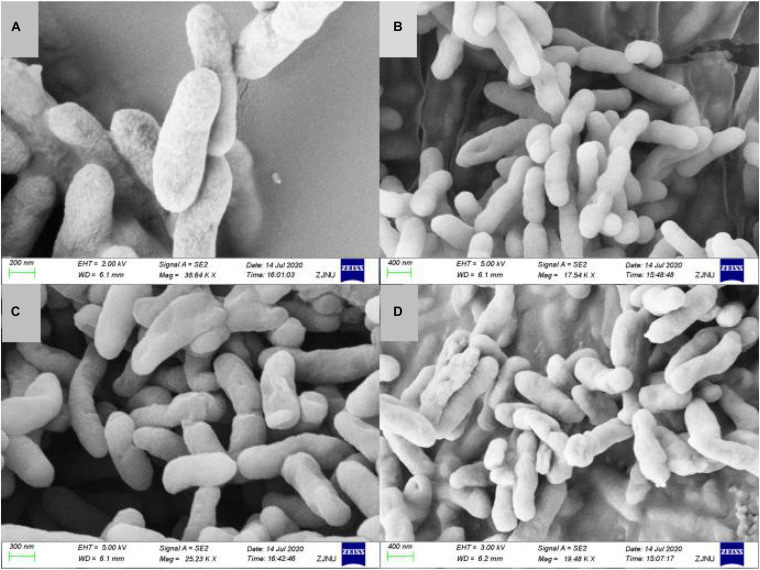
Scanning electron microscopy of *Xanthomonas oryzae* pv. *oryzae* (Xoo) treated with phosphate buffered solution (control) and various concentration of carbazomycin B for 4 h. **(A)** Control, **(B)** 1 × MIC carbazomycin B, **(C)** 2 × MIC carbazomycin B, and **(D)** 4 × MIC carbazomycin B.

Cell shape plays a critical role in regulating cellular functions in bacteria. Documenting carbazomycin B-induced morphology changes of Xoo could improve our understanding of the mechanisms involved. The morphological changes of Xoo, in response to carbazomycin B treatment were evaluated using scanning electron microscopy. The scanning electron micrograph revealed that the untreated cells of Xoo were still intact, plump, typically rod shaped, with a normal surface (Figure 3A). However, Xoo cells treated with carbazomycin B, treated for 4 h, showed irregular shape with sunken surfaces (Figures 3B–D). Furthermore, with the increasing concentration of carbazomycin B used, the cells deformed more severely and may even ruptured. These results indicated that carbazomycin B could damage the cell shape of Xoo, and the damaging effect was greater with an increased concentration.

### FT-IR Analysis

**FIGURE 4:**
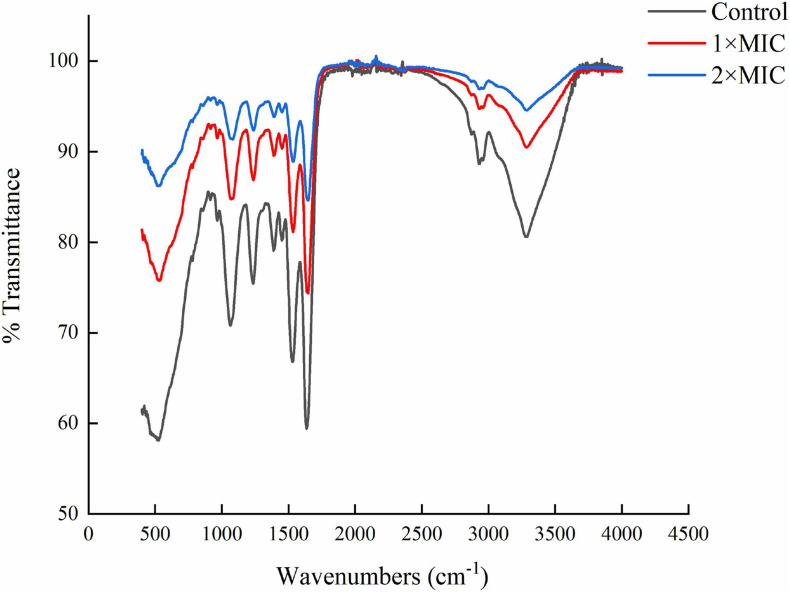
Fourier transform infrared spectroscopy (FTIR) showing the chemical modifications in *Xanthomonas oryzae* pv. *oryzae* (Xoo) after 4 h of carbzomycin B (1 × MIC and 2 × MIC) treatment. The black line indicates the shift in the IR spectra compared to untreated samples.

The carbazomycin B caused the morphology changes of Xoo, we further assessed the changes in components of the cell membrane induced by carbazomycin B. Through fourier transform infrared spectroscopy (FTIR) study, the Xoo cells were found to have led to a drastic shift in the absorbance peaks after carbazomycin B treatment (Figure 4 and Table 2). The major changes were in the range from 1,064 to 3,278 cm^–1^, as carabzomycin B interacted with the Xoo cell, which resulted in the shifting of the spectral band positions at 1064.24, 1234.36, 1388.36, 1528.77, 1636.55, 2929.72, and 3278.44 cm^–1^. The distinct spectral regions correspond to lipids (3,000–2,800 cm^–1^), proteins (1,800–1,480 cm^–1^), nucleic acids, and polysaccharides (1,250–900 cm^–1^) (Di Giambattista et al., 2011). The spectra clearly showed an indicating of chemical and physiological changes of the components of the cell membrane in Xoo.

**TABLE 2:** FT-IR vibrational peak assignment in response to carbazomycin B treatment.

**Definition of the spectral assignment**	**Classification**	**Peak variation (cm**^–^**^1^)**		
		**Control**	**1 × MIC**	**2 × MIC**
C-O, C-C str, C-O-H, C-O-C def of carbohydrates	Glycogen and nucleic acids	1064.24	1068.79	1081.48
P = O str (asym) of > PO_2_ phosphodiesters	Mainly nucleic acids	1234.36	1236.82	1237.94
C = O str (sym) of COO	amino acid side chains, fatty acids	1388.36	1389.18	1390.14
Amide II (protein N–H bend, C–N stretch)	α helices	1528.37	1533.94	1543.78
Amide I of β-pleated sheet structures	β-pleated sheet	1636.55	1647.42	1648.15
C-H str (asym) of > CH_2_	Mainly lipids	2929.72	2933.88	2935.29
O-H str of hydroxyl groups	Polysaccharides, proteins	3278.44	3283.42	3287.68

*Str, stretching; def, deformation; sym, symmetric; asym, antisymmetric.*

### Effect on the Hydrophobicity of Xoo Cells

**FIGURE 5:**
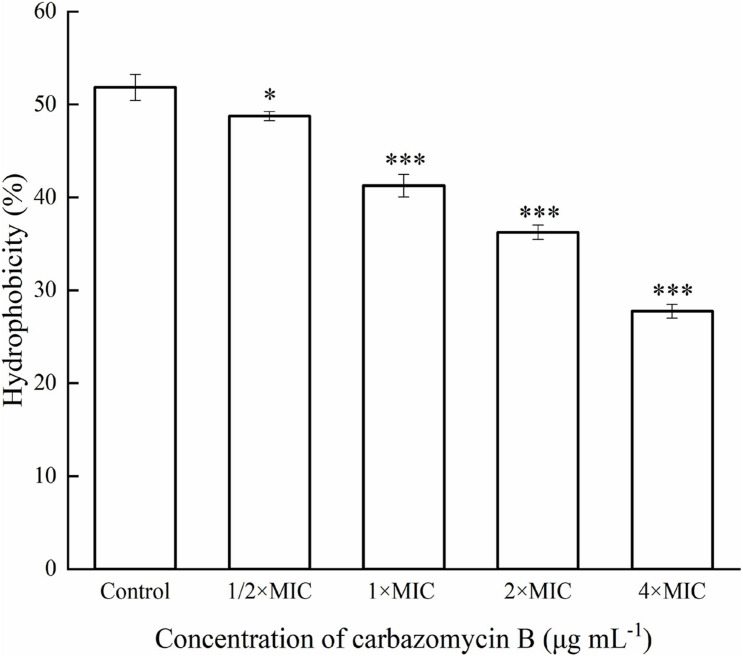
Hydrophobicity assay of *Xanthomonas oryzae* pv. *oryzae* (Xoo) treated with different concentration of carbazomycin B for 4 h. Results are means of three technical replicates and the bar indicates the standard deviation. Significant differences were determined by a *post hoc* test (^∗^*p* < 0.05 and ^∗∗∗^*p* < 0.001).

Cell surface hydrophobicity is related to the cell surface components, and it is a key factor in cell attachments. In this study, the Xoo cells treated with different carbazomycin B concentration all reduced hydrophobicity compared with the control group (Figure 5). The treatment with carbazomycin B at 1/2 × MIC, 1 × MIC, 2 × MIC, and 4 × MIC brought reductions of 3.08, 10.58, 15.58, and 24.08%, respectively. This indicated that carbazomycin B effectively reduced the hydrophobicity of Xoo.

### Determining the Influence of Carbazomycin B on the Metabolism of Xoo

**FIGURE 6:**
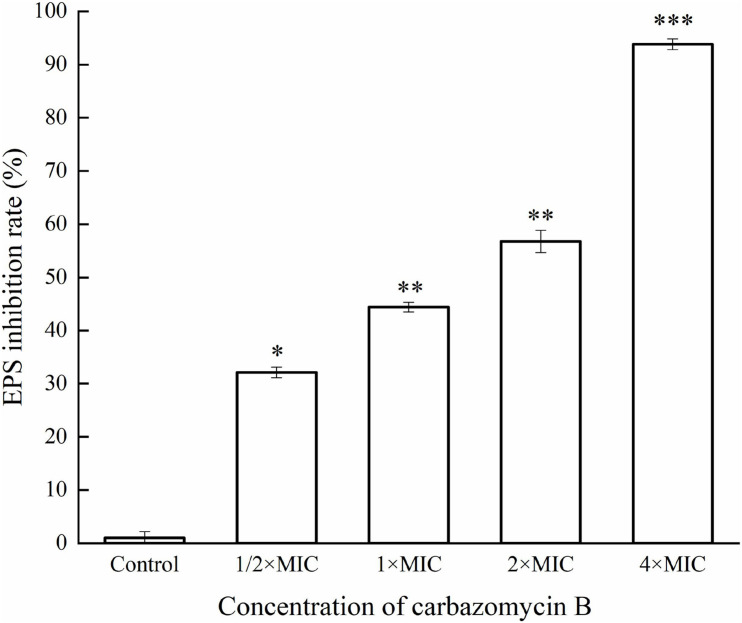
Inhibition rate of exopolysaccharide substance (EPS) production of *Xanthomonas oryzae* pv. *oryzae* (Xoo) by carbazomycin B treatment for 24 h. Results are means of three technical replicates and the bar indicates the standard deviation. Significant differences were determined by a *post hoc* test (^∗^*p* < 0.05, ^∗∗^*p* < 0.01, and ^∗∗∗^*p* < 0.001).

**FIGURE 7:**
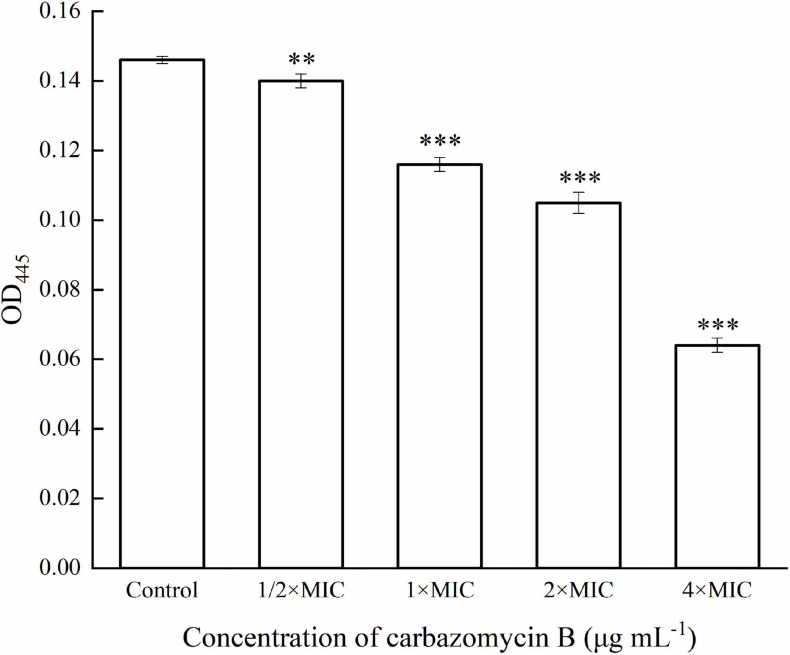
Reduction in xanthomonadin of *Xanthomonas oryzae* pv. *oryzae* (Xoo) after carbazomycin B treatment for 4 h. Results are means of three technical replicates and the bar indicates the standard deviation. Significant differences were determined by a *post hoc* test (^∗∗^*p* < 0.01 and ^∗∗∗^*p* < 0.001).

This study evaluated if carbazomycin B had any effect on the metabolism of Xoo. The results indicated that both xanthomonadin and EPS were essential for the growth and development of biofilm. As indicated in Figure 6, with increasing carbazomycin B concentration, the EPS for Xoo was greatly reduced. When the concentration of carbazomycin B increased from 1/2 × MIC, 1 × MIC, 2 × MIC to 4 × MIC, the reduction rates of EPS for Xoo were 32.1, 44.44, 56.79, 93.83%, respectively. Carbazomycin B was also found to block the production of xanthomonadin (Figure 7) in this study. The results showed that all concentrations of carbazomycin B significantly reduced the production of EPS for Xoo.

**FIGURE 8:**
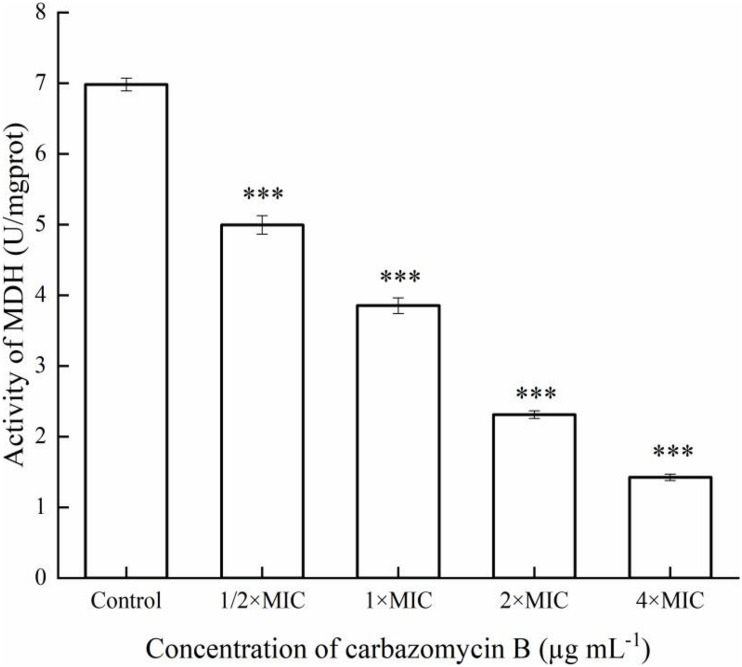
Effect of carbazomycin B on malate dehydrogenase (MDH) activity of *Xanthomonas oryzae* pv. *oryzae* (Xoo). The Xoo cells were treated with various concentration of carbazomycin B for 4 h. Results are means of three technical replicates and the bar indicates the standard deviation. Significant differences were determined by a *post hoc* test (^∗∗∗^*p* < 0.001).

Respiratory metabolism is an important manifestation of life activities for organisms, and its essence is due to the oxidative metabolism of sugars in cells. Once the oxidative metabolism of sugar is inhibited, the growth and reproduction of organisms will be blocked, or even cause death. Malate dehydrogenase (MDH), a key enzyme in the tricarboxylic acid (TCA) cycle, plays important metabolic roles in aerobic energy-producing pathways and malate shuttle (Han et al., 2014). The change of MDH activity changes can reflect the energy metabolism of cells. The results indicated that MDH activity of Xoo was induced after being treated with carbazomycin B (Figure 8). When the concentration of carbazomycin B increased from 1/2 × MIC, 1 × MIC, 2 × MIC to 4 × MIC, the reduction rates of MDH activity were 28.4, 44.8, 66.9, 79.6%, respectively. These results indicated that carbazomycin B significantly reduced MDH activity of Xoo, thus, negatively impacting the normal functioning of the TCA cycle.

**FIGURE 9:**
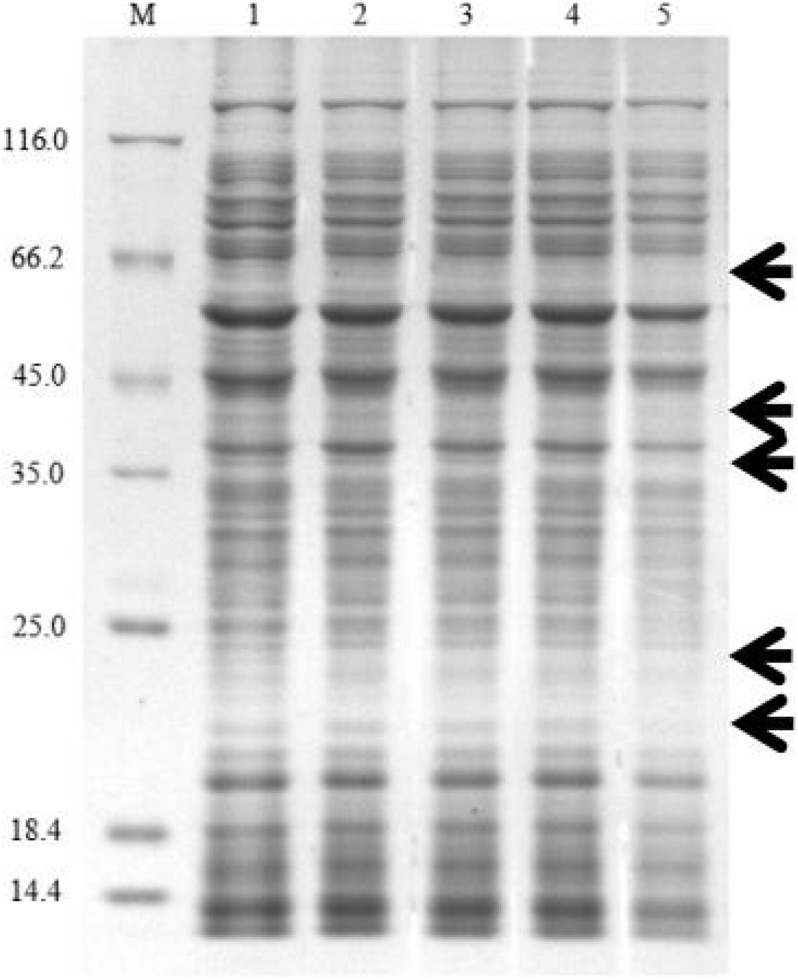
SDS-PAGE analysis of the change of total proteins in Xoo after treating with carbazomycin B for 4 h. Lane 1, PBS treatment as a control; lane 2, 1/2 × MIC carbazomycin B treatment for 4 h; lane 3, 1 × MIC carbazomycin B treatment for 4 h. lane 4, 2 × MIC carbazomycin B treatment for 4 h. lane 5, 4 × MIC carbazomycin B treatment for 4 h. The black arrows indicate lost protein bands.

Protein synthesis plays an essential role in cellular metabolism. This research showed that carbazomycin B could suppress the protein expression of Xoo. After treatment with 4 × MIC carbazomycin B for 4 h, the total protein profile of Xoo was decreased, with the loss of some bands in the SDS-PAGE analysis (Figure 9). The result indicated that carbazomycin B could exert its antibacterial effect by affecting the protein expression of Xoo.

## Discussion

The results from this preventative and curative effect experiments showed that the fermentation broth of Sr-63 had a remarkable curative activity against BLB, with a control effect of (95.35%) under the T1. The antibacterial substance, carbazomycin B, was isolated from the fermentation broth of Sr-63. The carbazomycin B was first isolated as the first antibiotics containing a carbazole nucleus, from *Streptoverticillium ehimense* H1051-MY 10 (Sakano et al., 1980). Carbazomycins B inhibited the growth of some kinds of phytopathogenic fungus (Sakano et al., 1980) and 5-lipoxygenase (Hook et al., 1990), with the weak antibacterial and antiyeast activities (Sakano et al., 1980), antimalarial activity (Karwehl et al., 2016), and also showed activity against lipid peroxidation in rat brain homogenate and DPPH (Kato et al., 1993). Carbazomycin A and B possess broad spectrum of biological activities due to their unusual carbazole skeleton. Carbazoles are a tricyclic skeleton that is frequently seen (Markad and Argade, 2014) as a core structure in biologically active compounds, which exhibit well-proven antitumor, antibiotic, antiviral, anti-HIV, anti-inflammatory, antimalarial, psychotropic, antihistaminic, antioxidative, and significant antituberculosis activities (Markad and Argade, 2014). The results showed the MIC of carbazomycin B against Xoo was 8 μg mL^–1^ while for bismerthiazol the MIC value was found to be 16 μg mL^–1^. However, to the authors’ knowledge, the biological activity of carbazomycin B resulting of inhibiting Xoo has not yet been reported.

Different types of components are present in extracellular polymeric substances: proteins, polysaccharides, DNA, RNA, peptidoglycan, lipids, phospholipids and other cell components (Sutherland, 2001), and the cell surface hydrophobicity was related to the cell surface structure like protein, lipoteichoic acid (Ma et al., 2015). Hydrophobicity is a key factor in cell attachment of bacteria to host tissue (Rosenberg et al., 1980), the stronger the surface hydrophobicity the bacteria had, the stronger resulting adhesion (Galán-Ladero et al., 2013). What’s more, the formation ability of biomembrane had a positive correlation with cell surface hydrophobicity (Bujdáková et al., 2013). In this study, the results indicated that carbazomycin B reduced the hydrophobicity of Xoo (Figure 5), which might be a consequence of the change in Xoo cell surface protein like structure, of lipoteichoic acid. The result was in agreement with the FTIR study, which showed the cell surface structure changes in Xoo after carbazomycin B treatment. This finding is in accordance with that of a previous study on the Xoo biofilm (Sahu et al., 2018). They demonstrated considerable changes of Xoo in chemical structures were detected by fourier transform infrared spectroscopy (FTIR), in response to niclosamide treatment. It is widely recognized that cell shape plays a critical role in regulating cellular functions in bacteria, SEM images (Figure 3) revealed noticeable deformation of Xoo after carbazomycin B treatment.

Microbial biofilms are considered as the most common form of growth for many microbials, and biofilm formation is a key virulence factor for a wide range of microorganisms. About 65% of all bacterial infections are associated with bacterial biofilms (Jamal et al., 2018), which are clusters of microorganisms that stick to non-biological surfaces as well as to surfaces on plants (roots) or in animals (epithelium) (Hall-Stoodley et al., 2004). The result from this study indicated that carbazomycin B may restrict the Xoo development of biofil (Figure 2). Xanthomonadins, the yellow membrane-bound pigments which were involved in biofilm formation (Poplawsky et al., 2000), has been linked to Xoo virulence (Yu et al., 2019). Extracellular polymeric substances (EPS) provide strength to the interaction of the microorganisms in the biofilm and format in the attachment stage of a biofilm to the surface (Jamal et al., 2018), and it is one of the most important virulence factors of Xoo (Jansson et al., 1975). Therefore, this study also examined the production of xanthomonadins and EPS. The data indicated that carbazomycin B reduced the production of both xanthomonadin (Figure 7) and EPS (Figure 6), which may be a reason the carbazomycin B reduced the virulence of Xoo (Figure 1). Collectively, the above results suggest that the reduction in the production of xanthomonadin and EPS, and the reduction of hydrophobicity, might be the possible cause of biofilm inhibition of Xoo by carbazomycin B. Similar studies on Xoo were reported earlier (Li et al., 2013; Wu et al., 2015; Sahu et al., 2018). Therefore, the antibacterial activities of carabzomucin B should be at least partly due to the damage caused in the cell membranes.

The present study measured the effects of the carbazomycin B on the activity of MDH in Xoo. The results demonstrated that the carbazomycin B could inhibit the pathway of respiratory metabolism, by inhibiting the tricarboxylic acid cycle. MDH is an important enzyme in the tricarboxylic acid cycle and the cell energy metabolism, and its activity changes can reflect the energy metabolism of cells. Protein synthesis plays an essential role in cellular metabolism. The most likely mode of action of Kocurin is a new thiazozyl peptide obtained from *Kocuria palustris* F-276,345, and the most likely mode of action is to inhibit bacterial growth by blocking its protein biosynthesis at the translation stage (Jakubiec-Krzesniak et al., 2018). The results suggest that protein expression may be suppressed by carbazomycin B (Figure 9), which may be related to the inhibition of the tricarboxylic acid cycle mentioned earlier. When Xoo cells were exposed to a high concentration of carbazomycin B, their respiration was disturbed and metabolism was blocked.

**FIGURE 10:**
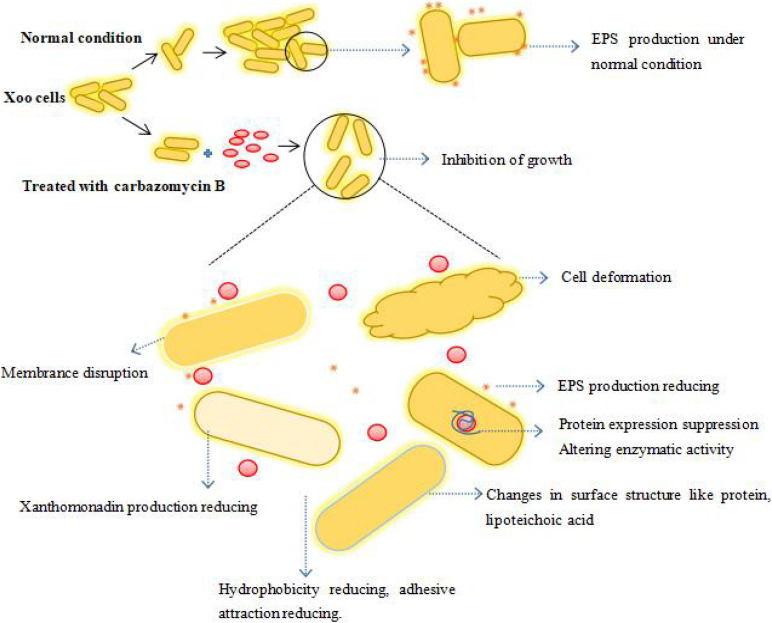
The proposed model depicting the potential mechanism of carbazomycin B against Xoo. The spherical red dot denotes the carbazomycin B.

Overall, carbazomycin B inhibited the growth of Xoo, it had a negative impact on the biomembrane formation, production of xanthomonadin and EPS, and the hydrophobicity of Xoo either directly or indirectly. The carbazomycin B also had a negative impact on normal pathogen metabolism. As a result, the pathogenicity of Xoo was decreased. Compounds belonging to a carbazole series have been identified as inhibitors of adenosine triphophatase (H^+^-ATPase) (Clausen et al., 2017) and tyrosinase (Ghani, 2019). From this standpoint, there is a high possibility that carbazomycin B exerts its antibacterial effects by inhibiting the activity of key enzymes (such as MDH), thereby causing all observed phenotypic changes (biofilm formation. hydrophobicity, protein expression, EPS, xanthomonadin, etc.). To conclude, we have summarized the whole of this study in an illustrative model. This depicts the possible mechanism by which carbazomycin B acts against Xoo (Figure 10). However, by taking into account the possible multiple impacts of carbazomycin B on Xoo, the involvement of other inhibitory mechanisms cannot be ruled out. Further research on transcriptome level changes might reveal more detailed mechanistic insights. This recognizes that carbazomycin B has not been previously used in agricultural management. In addition, many synthetic methods of carbazomycin B have been reported (Crich and Rumthao, 2004; Markad and Argade, 2014; Singh et al., 2019). These methods provide solutions for the industrial production of carbazomycin B. This could provide an attractive potential option for its use as an alternative to chemical bactericides to control BLB.

Biological control is an efficient strategy that might be applied in the management of plant diseases (Zeriouh et al., 2011). Microbial biological control agents protect crops from damage by diseases via different modes of action. They may induce resistance or prime enhanced resistance against infections by a pathogen in plant tissues (Conrath et al., 2015), competed for nutrients and space with pathogens (Spadaro and Droby, 2016), produced antimicrobial secondary metabolites (Raaijmakers and Mazzola, 2012), and so on. This study focused on the secondary metabolites produced by Sr-63 against Xoo. Further research is necessary to reveal alternative ways into how Sr-63 could be used as an important biological agent against BLB disease.

## Data Availability Statement

The original contributions presented in the study are included in the article/Supplementary Material, further inquiries can be directed to the corresponding author.

## Author Contributions

DJ and TS designed the research. DJ supervised the study. TS, JZ, and HL performed the experiments. TS and ZH analyzed the data. TS wrote the manuscript together with XG. All authors have read and agreed to the published version of the manuscript.

## Conflict of Interest

The authors declare that the research was conducted in the absence of any commercial or financial relationships that could be construed as a potential conflict of interest.
