# Supplementary material for: Inhibitory Effects of Carbazomycin B Produced by Streptomyces roseoverticillatus 63 Against Xanthomonas oryzae pv. oryzae
Source: Front Microbiol. 2021 Mar 24;12:616937. doi: 10.3389/fmicb.2021.616937 (PMC8024497; doi:10.3389/fmicb.2021.616937)
Supplement: Supplementary file 1 [file Data_Sheet_1.docx]

Supplementary Material

# Supplementary Figures


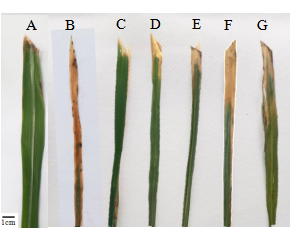


Supplementary figure S1 The lesion length suppression with different treatments after inoculation by *Xanthomonas oryzae* pv. *oryzae* (Xoo). A: Sprayed 500μg mL^-1^ of bismerthiazol after inoculation; B: Sprayed distilled water; C:Sprayed fermentation broth of Sr-63 after inoculation (T1); D:Sprayed fermentation broth of Sr-63 6 h later after inoculation (T2); E:Sprayed fermentation broth of Sr-63 12 h later after inoculation (T3); E:Sprayed fermentation broth of Sr-63 24 h laterafter inoculation (T4);


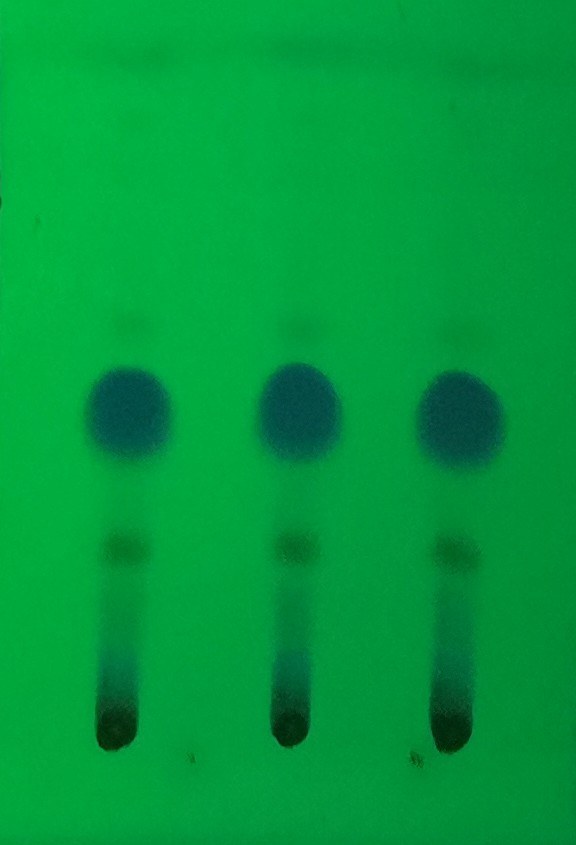


Supplementary figure S2 TLC analysis of carbazomycin B at 254 nm


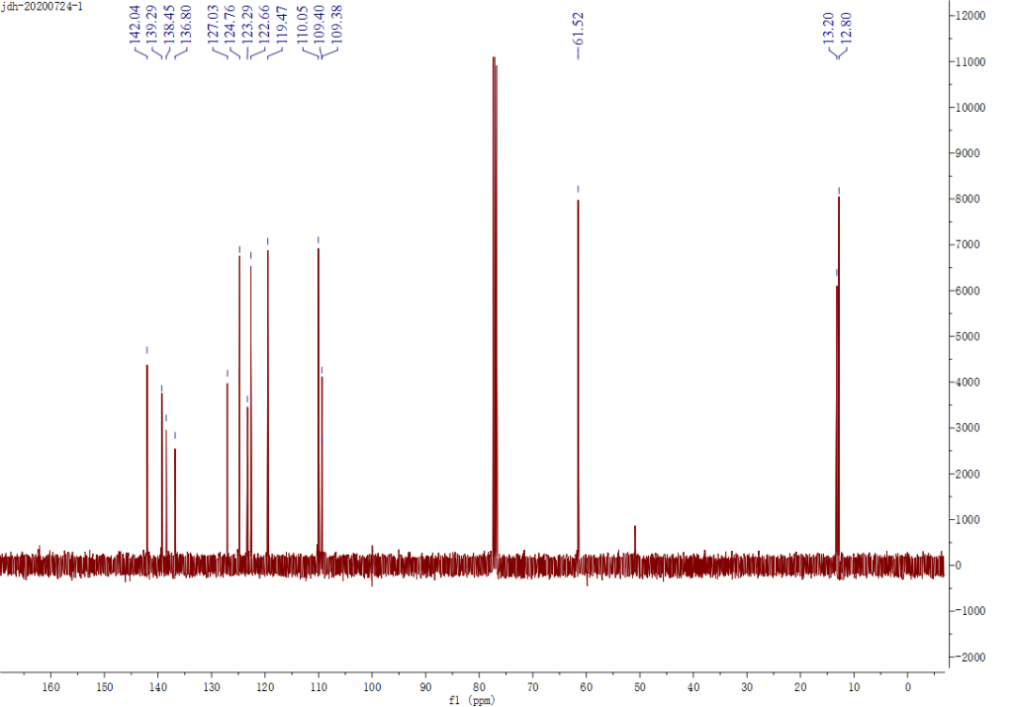


Supplementary figure S3 ^13^C NMR spectrum of carbazomycin B at 105 MHz in CDCl3


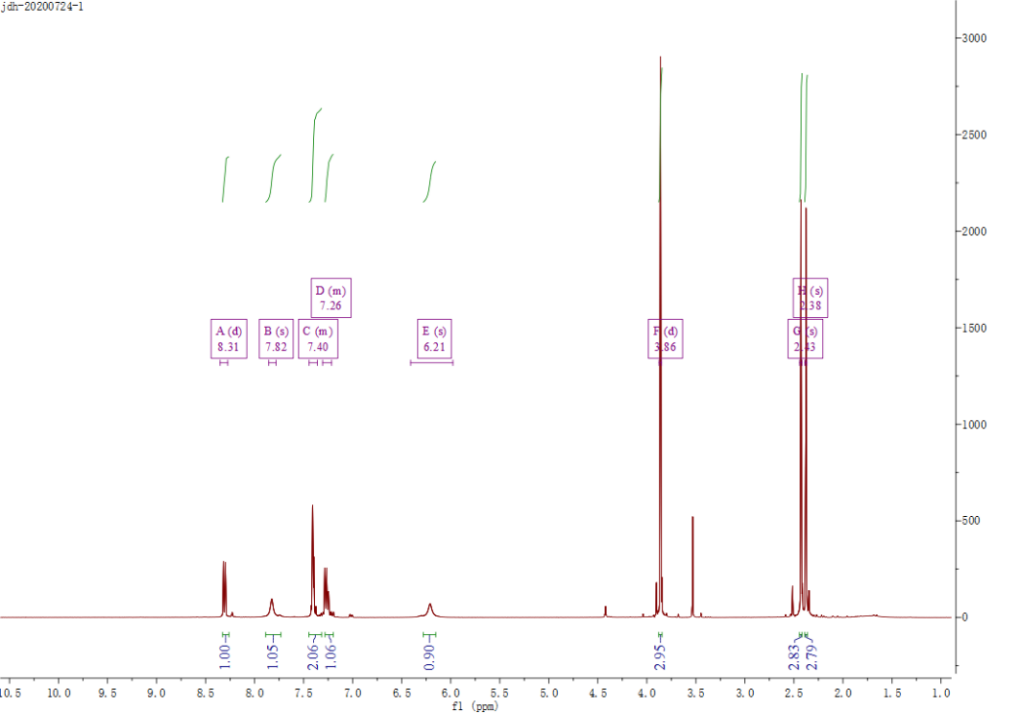


Supplementary figure S4 ^1^H NMR spectrum of compound 1 at 400 MHz in CDCl_3_


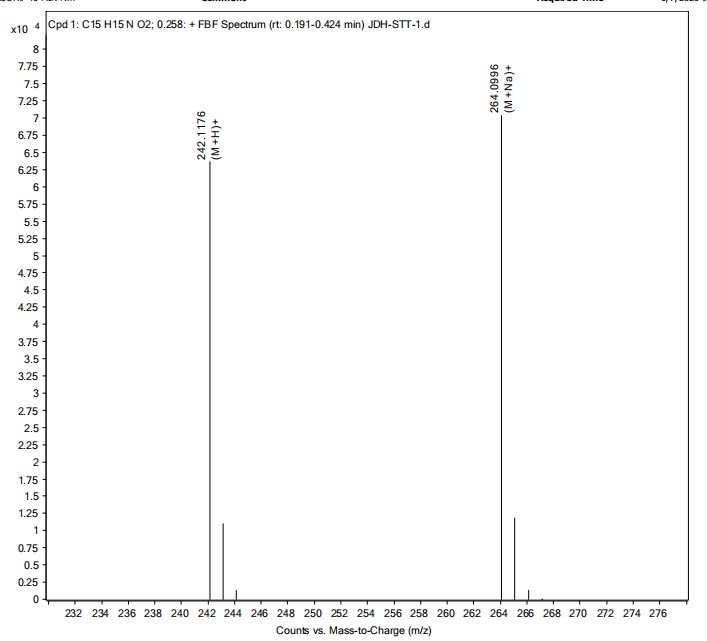


Supplementary figure S5 ESI mass spectra of carbazomycin B





Supplementary figure S6 Determination of MICs for carbazomicin B and bismerthiazol against Xoo by microdilution in 96-well plates. CK: Wells with nutrient broth containing 10^5^ CFU mL^−1^ of Xoo; NK: nutrient broth.
